# TicTimer Web: software for measuring tic suppression remotely

**DOI:** 10.12688/f1000research.26347.2

**Published:** 2021-03-03

**Authors:** Jonathan K. Black, Jonathan M. Koller, Kevin J. Black

**Affiliations:** 1 Department of Mechanical Engineering, Brigham Young University, Provo, Utah, 84602, USA; 2 Department of Psychiatry, Washington University in St. Louis, St. Louis, Missouri, 63110, USA; 3 Department of Radiology, Washington University in St. Louis, St. Louis, Missouri, 63110, USA; 4 Department of Neurology, Washington University in St. Louis, St. Louis, Missouri, 63110, USA; 5 Department of Neuroscience, Washington University in St. Louis, St. Louis, Missouri, 63110, USA

**Keywords:** tic disorders, Tourette syndrome, reward, reinforcement (psychology), software

## Abstract

Woods and Himle developed a standardized tic suppression paradigm (TSP) for the experimental setting, to quantify the effects of intentional tic suppression in Tourette syndrome. We previously provided a computer program to facilitate recording tic occurrence and to automate reward delivery during the several experimental conditions of the TSP. The present article describes a web-based program that performs the same functions. Implementing this program on the web allows research sessions to be performed remotely, in tandem with a video calling program. Relevant data for each session, such as the timing of tics and dispensed rewards, are stored in plain text files for later analysis. Expected applications include research on Tourette syndrome and related disorders.

## Introduction

One of the defining characteristics of tics, compared to some other abnormal movements, is that they can usually be suppressed with an effort of will for at least a brief interval ^1^. Woods and Himle developed an experimental measure of tic suppression, the tic suppression paradigm (TSP) ^2, 3^. In this paradigm, each participant is observed during several experimental conditions: usually baseline and differential reinforcement of zero-rate ticcing (DRO), and sometimes also verbal instruction to suppress tics and/or noncontingent reinforcement (NCR). The DRO condition replicates a behavior therapy long in clinical use, providing frequent rewards for absence of a problematic behavior, such as a reward token delivered after every 10 seconds without a tic. In the NCR condition, rewards are provided at a similar overall frequency as in the DRO condition, but at times unrelated to the timing of tics during the NCR session; Himle and colleagues used this approach to clarify the mechanism of the DRO condition’s tic reduction benefit ^4^.

The TSP has been used in a number of studies, producing several interesting results ^5, 6^. For instance, tic suppression measured early after the onset of a tic disorder predicts clinical outcome 6–12 months later ^7^. Additional studies using the TSP are being conducted in various research centers. In the course of conducting a longitudinal study of children with Provisional Tic Disorder ^8^, we found that the TSP required substantial investigator effort. That is, a tic expert must watch the session, “live” or on a video recording, and note the presence and timing of each tic. Furthermore, in the DRO and NCR conditions a second staff member must repeatedly signal a device to provide a reward for each predefined tic-free interval. To reduce this effort, we wrote a simple program to facilitate record keeping and reward delivery during research sessions ^9^. The expert observer pressed a button to record each tic observed, and in the DRO and NCR conditions the program delivered reward tokens at the appropriate times by connecting to a relay module that signaled a token dispenser box. This software improved convenience for the investigator and accuracy of record-keeping.

Because of enforced social distancing during the COVID 19 pandemic, the need arose for sessions to be performed remotely. A video calling program allowed us to observe the subjects, but we still needed a way to deliver rewards during the DRO and NCR conditions of the TSP. Previously, we had created a web-based program called TicTrainer for behavioral therapy ^10^, and we decided to expand the functionality of that program so it could be used for TSP research. The new program allows the same functionality as the previous, in-person TicTimer software, providing audiovisual rewards at appropriate intervals on a web browser viewed by the participant, based on the timing of tics recorded remotely by the investigator. We present the software here ^11^ to facilitate its use by others.

## Methods

### Implementation

TicTimer Web ^11^ uses the node.js server that was made for TicTrainer. It adopts that program’s structure, with user accounts for research subjects and an admin account for the rater. Details on account registration, data storage, and logging on were described previously ^10^.

A new field was added to user accounts so that a research ID (different from the ID used to log on) can be set for research subjects.

Sessions for TicTimer Web use separate but simultaneous connections to a server from a “user” and from a rater. TSP DRO sessions deliver rewards after every 10-s tic-free interval. To deliver rewards at the appropriate times, the user page checks in with the server periodically to see if it has been 10 seconds since the last tic was signaled by the rater. If it has not, the server responds with the time remaining until the next reward is due, and the user page uses that number, adjusted for the lag time of the round trip, to schedule when to check back with the server. Using this method, reward timing is synchronized so that rewards are delivered usually within 50 ms of the target time. The previous, in-person version of TicTimer used a token dispenser box to automatically deliver rewards at the appropriate times. In an attempt to approximate the user experience of the physical token dispenser, TicTimer Web delivers rewards by displaying coin images on the subject’s screen along with a chime sound.

At the end of each session, a summary is generated and appended at the bottom of the session log file, which is then archived with the date and time of the session in the filename.

### Operation

**Setup.** First, node.js is installed on the server. We used an Amazon EC2 instance, but the program can operate on any computer with node.js ( *e.g.* a laptop).

The researcher and subject need only a modern web browser to interface with TicTimer Web once the server is running. The browser must support JavaScript and HTML5. We have tested TicTimer Web with current versions of Chrome and Edge.

To perform sessions with TicTimer Web, the researcher creates a user account if needed, then if desired uses the admin interface to assign the user a research ID number to identify the subject for later data analysis.

**Use.** The researcher and subject sign in on their respective TicTimer session pages and the rater begins the session by selecting one of the four experimental conditions.

During a session, the rater watches the subject. We have used a separate video calling program for this observation, but a video camera or one-way mirror could be used for in-person visits. The rater records any observed tics by immediately pressing the “Tic Detected” button, the spacebar, or the letter “T”. If the session type includes rewards (DRO and NCR), they are dispensed appropriately. The session ends after the predetermined duration, or when the rater presses “End Session,” or when either rater or subject closes their browser window early.

For the NCR condition, the rater first chooses a log file previously created with the current subject, and rewards are delivered to the user at the same times (relative to the session start) that they were delivered in the specified session.

Archived session log files can be downloaded from the admin interface, or they can be copied over directly from the server itself.

## Use cases

The video file ( *Extended data*, Supplementary File 1) ^12^ demonstrates the operation of TicTimer Web ^11^ from a researcher’s perspective. The sessions performed here were test sessions with no human subjects being observed. *Extended data*, Supplementary Files 2–4 ^12^ are the session log files created in that video.

## Conclusions

The TicTimer Web ^11^ program allows for remote implementation of the TSP, while maintaining the benefits of earlier versions of the software ^9^: ease and accuracy of record keeping and automated reward delivery. TicTimer Web also simplifies the TSP, replacing a physical token dispenser box with any web browser, say a wireless tablet. While designed for our purposes in tic disorder research, TicTimer Web may find other uses. The most obvious of these may be for research on traditional habit disorders; for instance, hair pulling and skin picking appear in the “Obsessive-compulsive and related disorders” section of DSM-5 ^13^. TicTimer Web may also have clinical applications. These may include documenting suppression ability before and after treatment and investigations of the chaotic nature of tic timing ^14^, in addition to the previously described behavior therapy ^10^. 

Future modifications may include adding machine detection of tics, *e.g*. by audio-visual observation, surface EMG or accelerometry. Artificial intelligence and machine learning techniques may be able to use these inputs to recognize and mark the occurrence of specific tics. Such improvements would be quite welcome, as they might speed tic research or even allow automated behavior therapy. However, these methods are thus far difficult to reduce to practice; separating tics from normal adventitious movements is not trivial, and the wide variety of observed tics defies a unitary definition in terms of elementary movement features and timing.

## Data availability

### Underlying data

All data underlying the results are available as part of the article and no additional source data are required.

### Extended data

Zenodo: TicTimer Web: software for measuring tic suppression remotely: Supplementary Files. http://doi.org/10.5281/zenodo.4023134 ^12^.

This project contains the following extended data:

- ttw_demo.mp4. (Supplementary File 1: Video Demonstration of Operation. A video demonstrating how to operate TicTimer Web from the researcher’s perspective.)
- au5_20200622-153043_baseline.ttsd. (Supplementary File 2: Sample Log, baseline. Log file for the baseline session performed during the video demonstration.)
- au5_20200622-153125_DRZ.ttsd. (Supplementary File 3: Sample Log, DRO. Log file for the DRO session performed during the video demonstration.)
- au5_20200622-153214_NCR.ttsd. (Supplementary File 4: Sample Log, NCR. Log file for the NCR session performed during the video demonstration.)

License: MIT License.

## Software availability

The source code for TicTimer Web is available at: https://github.com/jonkb/TicTrainer-node.

Archived source code at time of publication: https://doi.org/10.5281/zenodo.3990474 ^11^.

License: MIT License.
