# Supplementary material for: TicTimer Web: software for measuring tic suppression remotely
Source: F1000Res. 2021 Mar 3;9:1264. Originally published 2020 Oct 22. [Version 2] doi: 10.12688/f1000research.26347.2 (PMC7993402; doi:10.12688/f1000research.26347.2)
Supplement: Peer review history

# Peer review: Reviewer response for version 2

**DOI:** 10.5256/f1000research.54969.r80760

**Published:** 2021-03-23

**Authors:** Matthew W. Specht, Jennifer Schild

**Affiliations:** 1 Weill Cornell Medicine, New York City, NY, USA; 2 Suffolk University, Boston, MA, USA

The authors appear to have responded to our prior suggested edits and comments, we have no further comments at this time.

Are the conclusions about the tool and its performance adequately supported by the findings presented in the article?

Partly

Is the rationale for developing the new software tool clearly explained?

Partly

Is the description of the software tool technically sound?

Yes

Are sufficient details of the code, methods and analysis (if applicable) provided to allow replication of the software development and its use by others?

Yes

Is sufficient information provided to allow interpretation of the expected output datasets and any results generated using the tool?

Partly

Reviewer Expertise:

I have 15 years of research regarding Tourette's disorder and have utilized the tic detector paradigm in my own research.

We confirm that we have read this submission and believe that we have an appropriate level of expertise to confirm that it is of an acceptable scientific standard.

# Peer review: Reviewer response for version 1

**DOI:** 10.5256/f1000research.29084.r77056

**Published:** 2021-02-17

**Authors:** Valerie Brandt

**Affiliations:** 1 Department of Psychology, Centre for Innovation in Mental Health, University of Southampton, Southampton, UK

The report describes the development of TicTimer Web, a nifty software that can be used to record tics, tic timings, and distribute reinforcement tokens during online experiments are therapeutic sessions. The authors describe that they adapted the TicTrainer programme to an online version that allows patients to login to a user account and therapists / tic raters to login as administrators. User and rater are then connected to the server simultaneously. The tic rater can record tics via button press and the software stores the timing of that tic. After every tic-free 10 second interval, the software delivers a reward. The software also delivers a summary at the end of the session.

 In order for the rater to watch tics, a separate programme, or a webcam can be used. This allows an accurate reward delivery, even remotely.

 The source code of TicTimer is available on GitHub, the link is provided in the manuscript. Furthermore, an explanatory video shows how TicTimer can be used. The video is simple and clear and provides step-by-step explanations. Examples of demo files are also provided on Zenodo.

 The manuscript is clearly written, the software is demonstrated in a video, examples and the source code are provided.

 I have no suggestions for improvement.

Are the conclusions about the tool and its performance adequately supported by the findings presented in the article?

Yes

Is the rationale for developing the new software tool clearly explained?

Yes

Is the description of the software tool technically sound?

Yes

Are sufficient details of the code, methods and analysis (if applicable) provided to allow replication of the software development and its use by others?

Yes

Is sufficient information provided to allow interpretation of the expected output datasets and any results generated using the tool?

Yes

Reviewer Expertise:

Tic disorders.

I confirm that I have read this submission and believe that I have an appropriate level of expertise to confirm that it is of an acceptable scientific standard.

# Response

**Published:** 2021-02-22

**Authors:** Kevin J Black

**Affiliations:** Department of Psychiatry, Washington University in St Louis, USA

We thank Prof. Brandt for her kind comments.

# Peer review: Reviewer response for version 1

**DOI:** 10.5256/f1000research.29084.r73543

**Published:** 2021-01-20

**Authors:** Matthew W. Specht, Jennifer Schild

**Affiliations:** 1 Suffolk University, Boston, MA, USA; 2 Weill Cornell Medicine, New York City, NY, USA

This paper describes a timely and valuable tool for assessing tic suppression remotely in clinical and research settings. This web-based program is based on Woods and Himle’s standardized tic suppression paradigm (TSP) and performs the same function as a computer program previously developed by the authors to record tics and partially automate delivery of rewards. This paper is particularly useful during this time in which remote data collection for research is relevant and necessary, and it provides a helpful overview of a program that can be easily understood by other tic researchers to apply in their own research. The paper could benefit from elaboration in multiple areas to help readers better understand the broader relevance of TSP as well as the research utility of TicTimer Web. Elaboration in the introduction section, as well as a more detailed description of potential applications of this software and how this software helps to overcome previous limitations of the TSP are needed. We recommend that this article be revised and resubmitted. Below are some suggestions for the authors to consider.

1. Please provide a clearer, more detailed explanation of the broad importance of TSP and related concepts in the introduction section. This will be helpful for readers who are less familiar with tic suppression to understand what insights this paradigm have yielded and how this software program could be beneficial to future investigation. For example, the TSP is described as requiring “substantial investigator effort,” but it is not clear what exactly is entailed. Those who used the old tic-detector boxes know how laborious running a participant was, but those less familiar will not appreciate the leap forward TicTimer Web provides.
2. A short description of the TicTrainer web-based program is also needed in the introduction section. This will be helpful for orienting the reader to the new program that is described in this article, especially considering how similar this older program is to TicTimer Web.
3. In the methods section, there is interchangeable usage of the term “reward” and “award.” The word “awards” in paragraph 3 of the implementation subsection of the methods should be replaced with “rewards.” 
4. In the methods section, elaboration is needed on what each experimental condition is (e.g., differential reinforcement of zero-rate ticcing (DRO), noncontingent reinforcement (NCR). Alternatively, this can be elaborated upon in the introduction section as it is also mentioned there.
5. A separate section before the conclusion discussing the strengths and future applications of this software would be useful. This is somewhat addressed in the conclusion section, but further elaboration in a devoted section would do a better job of highlighting the potential of this software program.
6. Next steps: It would be helpful to provide another sentence or two regarding the potential benefits of using artificial intelligence (AI) and machine learning (ML) to do this job as well as barriers to taking this approach. 

Overall, this paper provides a concise and useful overview of a web-based program for remote implementation of the TSP. With some clarification in the areas highlighted above, this paper offers a useful contribution to tic research and is especially applicable given the current relevance of remote methods for data collection. We strongly encourage the authors to revise and resubmit this manuscript for consideration.

Are the conclusions about the tool and its performance adequately supported by the findings presented in the article?

Partly

Is the rationale for developing the new software tool clearly explained?

Partly

Is the description of the software tool technically sound?

Yes

Are sufficient details of the code, methods and analysis (if applicable) provided to allow replication of the software development and its use by others?

Yes

Is sufficient information provided to allow interpretation of the expected output datasets and any results generated using the tool?

Partly

Reviewer Expertise:

I have 15 years of research regarding Tourette's disorder and have utilized the tic detector paradigm in my own research.

We confirm that we have read this submission and believe that we have an appropriate level of expertise to confirm that it is of an acceptable scientific standard, however we have significant reservations, as outlined above.

# Response

**Published:** 2021-02-22

**Authors:** Kevin J Black

**Affiliations:** Department of Psychiatry, Washington University in St Louis, USA

We thank Ms. Schild and Dr. Specht for their thoughtful review, and will edit the report as they suggest.

# Response

**Published:** 2021-02-24

**Authors:** Kevin J Black

**Affiliations:** Department of Psychiatry, Washington University in St Louis, USA

**Author responses are below, marked by the > sign and bold face, intercalated between sections of the original review.**

 APPROVED WITH RESERVATIONS

 This paper describes a timely and valuable tool for assessing tic suppression remotely in clinical and research settings. This web-based program is based on Woods and Himle’s standardized tic suppression paradigm (TSP) and performs the same function as a computer program previously developed by the authors to record tics and partially automate delivery of rewards. This paper is particularly useful during this time in which remote data collection for research is relevant and necessary, and it provides a helpful overview of a program that can be easily understood by other tic researchers to apply in their own research.

**> Thank you for these positive comments.**

 The paper could benefit from elaboration in multiple areas to help readers better understand the broader relevance of TSP as well as the research utility of TicTimer Web. Elaboration in the introduction section, as well as a more detailed description of potential applications of this software and how this software helps to overcome previous limitations of the TSP are needed. We recommend that this article be revised and resubmitted. Below are some suggestions for the authors to consider.

1. Please provide a clearer, more detailed explanation of the broad importance of TSP and related concepts in the introduction section. This will be helpful for readers who are less familiar with tic suppression to understand what insights this paradigm have yielded and how this software program could be beneficial to future investigation. For example, the TSP is described as requiring “substantial investigator effort,” but it is not clear what exactly is entailed. Those who used the old tic-detector boxes know how laborious running a participant was, but those less familiar will not appreciate the leap forward TicTimer Web provides.

**> Done (first 2 paragraphs).**

  

1. A short description of the TicTrainer web-based program is also needed in the introduction section. This will be helpful for orienting the reader to the new program that is described in this article, especially considering how similar this older program is to TicTimer Web.

**> Done (last paragraph of Intro).**

  

1. In the methods section, there is interchangeable usage of the term “reward” and “award.” The word “awards” in paragraph 3 of the implementation subsection of the methods should be replaced with “rewards.” 

**> Thank you. Corrected.**

  

1. In the methods section, elaboration is needed on what each experimental condition is (e.g., differential reinforcement of zero-rate ticcing (DRO), noncontingent reinforcement (NCR). Alternatively, this can be elaborated upon in the introduction section as it is also mentioned there.

**> Done (in first paragraph of Intro).**

  

1. A separate section before the conclusion discussing the strengths and future applications of this software would be useful. This is somewhat addressed in the conclusion section, but further elaboration in a devoted section would do a better job of highlighting the potential of this software program.

**> I elaborate on these in the first paragraph of the revised Conclusions section.**

  

1. Next steps: It would be helpful to provide another sentence or two regarding the potential benefits of using artificial intelligence (AI) and machine learning (ML) to do this job as well as barriers to taking this approach. 

**> Done (last paragraph of Conclusions).**

 Overall, this paper provides a concise and useful overview of a web-based program for remote implementation of the TSP. With some clarification in the areas highlighted above, this paper offers a useful contribution to tic research and is especially applicable given the current relevance of remote methods for data collection. We strongly encourage the authors to revise and resubmit this manuscript for consideration.

**> Thank you.**
